# Daily fluctuations in COVID-19 infection rates under Tokyo’s epidemic prevention measures – new evidence from adaptive Fourier decomposition

**DOI:** 10.3389/fpubh.2023.1245572

**Published:** 2023-12-14

**Authors:** Guibin Lu, Zifeng Yang, Wei Qu, Tao Qian, Zige Liu, Wei He, Zhijie Lin, Chitin Hon

**Affiliations:** ^1^Department of Engineering Science, Faculty of Innovation Engineering, Macau University of Science and Technology, Taipa, Macao SAR, China; ^2^National Clinical Research Center for Respiratory Disease, State Key Laboratory of Respiratory Disease, Guangzhou Institute of Respiratory Health, The First Affiliated Hospital of Guangzhou Medical University, Guangzhou, Guangdong, China; ^3^Guangzhou Laboratory, Guangzhou, Guangdong, China; ^4^Respiratory Disease AI Laboratory on Epidemic Intelligence and Medical Big Data Instrument Applications, Department of Engineering Science, Faculty of Innovation Engineering, Macau University of Science and Technology, Taipa, Macao SAR, China; ^5^Guangzhou Key Laboratory for Clinical Rapid Diagnosis and Early Warning of Infectious Diseases, KingMed School of Laboratory Medicine, Guangzhou Medical University, Guangzhou, China; ^6^College of Sciences, China Jiliang University, Hangzhou, China; ^7^Macau Center for Mathematical Sciences, Macau University of Science and Technology, Macau, China

**Keywords:** adaptive Fourier decomposition, COVID-19, daily fluctuation, epidemic prevention policies, Tokyo pandemic

## Abstract

**Background:**

The COVID-19 pandemic has witnessed widespread infections and variants. Particularly, Tokyo faced the challenge of seven waves of COVID-19, during which government interventions played a pivotal role. Therefore, gaining a comprehensive understanding of government control measures is of paramount importance, which is beneficial for health authorities in the policy development process.

**Method:**

Our study analysis the daily change data of the daily COVID-19 infection count in Tokyo from January 16, 2020 to September 30, 2022. We utilized adaptive Fourier decomposition (AFD) for analyzing the temporal trends within COVID-19 data. It extends the conventional AFD approach by constructing new components base on multiple individual components at various time-frequency scales. Furthermore, we conducted Pearson correlation assessments of the first to third-order synthesis results, along with comparative analyses against other signal analysis techniques. Ultimately, these new components are integrated with policy data spanning different time periods for a comprehensive analysis.

**Result:**

The analysis of daily COVID-19 data in Tokyo using AFD reveals how various government policies impacted infection rates across seven distinct fluctuation periods. In the decomposition results, the reduction of business hours policy correlated with high-frequency components in the first four waves, while the low-frequency components for the sixth wave suggested a decline in its relevance. The vaccination policy initially displayed a mid-frequency correlation with the fifth wave and continued with a low-frequency correlation in the last wave. Moreover, our statistical analysis (value of *p* < 0.05) demonstrated that 75% of the third-order AFD components exhibited significant positive correlations with the original infections, while the correlation coefficients of most components in EMD and VMD did not attain significance.

**Conclusion:**

In the time-frequency domain, AFD demonstrates superior performance compared to EMD and VMD in capturing crucial data related to epidemic control measures. The variations in daily COVID-19 infection counts during these seven periods under various policies are evident in distinct third-order AFD components. These findings guide the formulation of future public health policies and social measures.

## Introduction

The World Health Organization (WHO) officially recognized the Coronavirus Disease 2019 (COVID-19) as a public health emergency of international concern, reflecting the global gravity of the pandemic. This declaration was made on January 30, 2020, in response to the escalating threat posed by this novel coronavirus outbreak (1, 2). On January 22, 2023, the reported global infection cases had exceeded hundreds of millions due to cumulative mutations, which notable variants include Alpha (B.1.1.7), Delta (B.1.617), and Omicron (B.1.1.529) (3, 4, 5, 6).

As of October 2022, Japan’s COVID-19 epidemiological landscape had seen seven distinct waves. Each was mitigated through a combination of public health measures, social interventions, and vaccination campaigns. Nevertheless, the recurrence of social activities and the emergence of new viral variants frequently precipitated fresh outbreaks. In Tokyo, Japan’s capital, the daily count of COVID-19 cases significantly informs the development of preventative policies and profoundly affects the country’s disease control efforts. Consequently, higher daily infection counts often precipitate more stringent prevention measures. Thus, analyzing daily infection trends can not only enhance residents’ pandemic preparedness but also offer valuable insights for policymakers striving to optimize disease prevention and control strategies.

The existing body of literature largely resorts to econometric approaches, such as SI, SIR, SIRS, and SEIR models, to scrutinize the day-to-day fluctuations in COVID-19 cases (7–13). Most of these studies primarily focus on the time-domain attributes and the linear modeling of daily infection tallies. Nonetheless, the daily COVID-19 case count encapsulates intricate non-linear data, bearing frequency-domain properties. Many previous studies have used EMD to conduct analysis related to the spread of COVID-19, among which there is a non-linear data analysis study on the number of daily infections with the implementation of public health measures (14–20). EMD is a class due to the consideration of frequency domain information, but the EMD-based model can only extract the time-frequency information of the time series, and there are still shortcomings such as scale aliasing (21). Since EMD is based on local features of the data, its decomposition components can be derived adaptively (22). However, this approach has no clear mathematical explanation, making its decomposition results difficult to understand and interpret. Furthermore, the analytic phase function of IMF is not monotonic in some cases (23). In other words, a physically meaningful instantaneous frequency for IMF analysis cannot be defined in general, which will affect the time-frequency analysis of IMF (22, 24–26). To address these limitations, the adaptive Fourier decomposition (AFD) method was introduced by Qian et al. (25, 27, 28). Providing a rigorous mathematical foundation that EMD lacks (25, 28), AFD proves to be more apt for analyzing daily fluctuations in the number of COVID-19 infections.

In this study, we explore the oscillations of daily COVID-19 infection counts in Tokyo using a novel AFD time-frequency model. The AFD model effectively avoids the scale aliasing phenomenon and employs transient time-frequency distribution to identify periods of significant fluctuation. Building upon the model’s original transient time-frequency distribution, we innovatively reconstruct the time series of daily infections by extracting components across different time-frequency scales. Moreover, we contrast the AFD’s decomposition method with two renowned time-frequency analysis techniques, EMD and VMD.

## Methods

### Adaptive Fourier decomposition

Adaptive Fourier decomposition (AFD) is an algorithm used to process discrete time signals by dynamically adjusting the frequency resolution to enhance signal processing efficiency. In contrast, Empirical Mode Decomposition (EMD), another signal processing technique, can yield decomposition results with negative phase derivatives in practical applications, lacking a clear mathematical explanation and making it challenging to interpret (29). AFD, on the other hand, surpasses other decomposition methods by adaptively generating an input-dependent basis, thereby achieving efficient decomposition. It separates the signal into multiple individual components, each consisting solely of non-negative analytic phase derivatives, ranging from high-energy modes to low-energy modes, with a rigorous mathematical foundation (28). From the overall data curve of daily COVID-19 infection, it can be seen that the spread of COVID-19 is nonlinear, and AFD can decompose the nonlinear composite signals collected in the real world, so AFD can be used to analyze the daily COVID-19 infection count under the policy (28, 29).

The Takenaka-Malmquist system, ${\left\{B_{n}\right\}}_{n=1}^{\infty }$ , is used as the basis elements of the AFD where:


(1) $$B_{n}\left(e^{jt}\right)=\frac{\sqrt{1-{\left|a_{n}\right|}^{2}}}{1-{\bar{a}}_{n}e^{jt}}\overset{n-1}{\underset{k=1}{\prod }}\frac{e^{jt}-a_{k}}{1-{\bar{a}}_{k}e^{jt}}\text{,}$$

*a_n_* ∈ D, D ⊆ C, *n* = 1,2,…, with C representing the complex plane (29). Due to the fact that the characteristics of $B_{n}\left(e^{jt}\right)$ are related to $a_{n}$ , the main task of AFD is to find such arrays $\left\{a_{1},a_{2},\cdots ,a_{n}\right\}$ , so that all decomposition components only contain phase derivatives with high decomposition efficiency and physical significance.

In the AFD algorithm, all single components are sequentially extracted from high-energy mode to low-energy mode. In order to easily find the energy relationship, reduce the remainder ${G_{n}}^{’}s$ and define ${R_{n-1}}^{’}s$ with their corresponding standard remainder (21):


(2) $$G_{n}\left(e^{jt}\right)=R_{n-1}\left(e^{jt}\right)\overset{n-1}{\underset{l=1}{\prod }}\frac{1-{\bar{a}}_{l}e^{jt}}{e^{jt}-a_{l}}$$

So $G\left(t\right)$ can be represented by the simplified remainder ${G_{n}}^{’}s$ :


(3) $$G\left(t\right)=\overset{N}{\underset{n=1}{\sum }}\langle G_{n},e_{\left\{a_{n}\right\}}\rangle B_{n}\left(e^{jt}\right)+G_{N+1}\left(e^{jt}\right)\overset{N}{\underset{n=1}{\prod }}\frac{e^{jt}-a_{n}}{1-{\bar{a}}_{l}e^{jt}}$$

$e_{\left\{a_{n}\right\}}\left(e^{jt}\right)$ is called the evaluator at $a_{n}$ which constitute a dictionary of the complex Hardy space $H^{2}\left(D\right)$ (30).


(4) $$e_{\left\{a_{n}\right\}}\left(e^{jt}\right)=\frac{\sqrt{1-{\left|a_{n}\right|}^{2}}}{1-{\bar{a}}_{n}e^{jt}}$$

The complex Hardy space in the unit circle D is defined as follows:


$$\begin{array}{l} H^{2}\left(D\right)\\ =\left\{f:D\to C:f\;\mathrm{is}\;\mathrm{holomorphic},\;\mathrm{and}{\left\|f\right\|}_{2}^{2}\underline{\;\underline{\Delta }}\underset{1>r\geq 0}{\sup}\overset{2\pi }{\underset{0}{\int }}{\left|f\left(re^{jt}\right)\right|}^{2}dt<\infty \right\}\text{.} \end{array}$$

According to (3), the energy of $G\left(t\right)$ can be calculated by (21).


(5) $$∥G\left(t\right){∥}^{2}=\overset{N}{\underset{n=1}{\sum }}{\left|\langle G_{n},e_{\left\{a_{n}\right\}}\rangle \right|}^{2}+∥G_{N+1}\left(e^{jt}\right){∥}^{2}$$

In order to minimize the energy of the standard remainder $∥G_{N+1}\left(e^{jt}\right){∥}^{2}$ , the maximum projection principle shown in (6) is used to find $a_{n}$ that can generate the maximum ${\left|\langle G_{n},e_{\left\{a_{n}\right\}}\rangle \right|}^{2}$ for each step n (21).


(6) $$a_{n}=\arg\;\max\left\{{\left|\langle G_{n},e_{\left\{a_{n}\right\}}\rangle \right|}^{2}:a_{n}\in D\right\}$$

From this algorithm, there are significant differences between the AFD method and traditional decomposition methods. AFD decomposes signals based on their energy distribution, making it suitable for separating signals with overlapping frequencies.

### New components construction based on adaptive Fourier decomposition results

AFD has different variants, including core AFD, unwinding AFD, cyclic AFD, and random AFD (30–34), and has been applied in various fields (35–37). When performing signal decomposition of large-scale data, in order to ensure sufficient decomposition, more layers of decomposition results are usually set. This inevitably leads to redundant data generated by signal decomposition. By merging partial decomposition results, the risk of data errors can be reduced and the reliability of the data can be improved. Therefore, we attempted to construct new components base on the decomposition results:


(7) $$D_{p}\left(q\right)=\overset{pq}{\underset{m=pq-q+1}{\sum }}C_{m}B_{m}\left(e^{jt}\right)$$

$$\mathrm{where}\;C_{m}=$$

$$\langle G_{m},e_{\left\{a_{m}\right\}}\rangle$$

$$\mathrm{and}\;B_{m}\left(e^{jt}\right)=$$

$$\frac{\sqrt{1-{\left|a_{m}\right|}^{2}}}{1-{\bar{a}}_{m}e^{jt}}\overset{m-1}{\underset{k=1}{\prod }}\frac{e^{jt}-a_{k}}{1-{\bar{a}}_{k}e^{jt}}\text{.}$$

(8) $$F_{q}=\overset{r}{\underset{p=1}{\sum }}C_{p}\left(q\right)$$

### The Pearson correlation coefficient

Correlation analysis is a statistical method used to evaluate the strength and direction of the linear relationship between two quantitative variables. The result of the analysis is expressed as a correlation coefficient, commonly denoted as ‘r’. This coefficient can range between −1 and 1, where −1 indicates a perfect negative linear relationship, 1 indicates a perfect positive linear relationship, and 0 indicates no linear relationship (38). In correlation analysis, the value of *p* assesses the probability of observing a particular correlation coefficient, r, assuming no true correlation exists in the population. A low value of *p* suggests the correlation is statistically significant (39).

The Pearson correlation coefficient r between two variables x and y is defined as:


(9) $$r=\frac{\sum_{i=1}^{n}\left(x_{i}-\bar{x}\right)\left(y_{i}-\bar{y}\right)}{\sqrt{\sum_{i=1}^{n}{\left(x_{i}-\bar{x}\right)}^{2}\sqrt{\sum_{i=1}^{n}{\left(y_{i}-\bar{y}\right)}^{2}}}}$$

where $x_{i}$ and $y_{i}$ are the $i^{th}$ data points, $\bar{x}$ and $\bar{y}$ are the means of X and Y respectively, and n is the number of data points.

### Sample and data

Our empirical examination is grounded in the fluctuation of the daily COVID-19 infection count in the Tokyo epidemic prevention and control policy from January 16, 2020 to September 30, 2022. Fraser et al. (40) scrutinized the spread of COVID-19 across Japan’s 47 prefectures, discovering significant variation attributable to different social ties. Consequently, the propagation of COVID-19 diverges across regions under varying circumstances.

Parallelly, Watanabe et al. (41) suggested that during Japan’s COVID-19 outbreak, the government-implemented public health measures exerted an interventionist effect, while the social measures catalyzed an informational effect that altered people’s behavior. Tokyo, being Japan’s nexus of politics, economy, culture, and transportation—housing the largest population among all prefectures and boasting the country’s highest population density—is particularly representative. Moreover, Tokyo is widely recognized for possessing the most comprehensive medical resources, spanning high-end medical equipment, advanced technology, and quality medical services (37).

**Figure 1:**
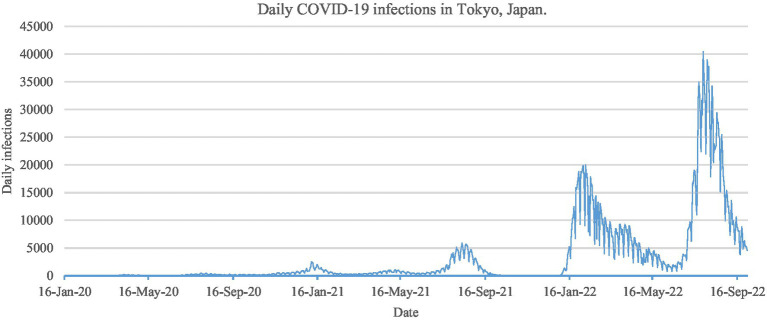
Daily COVID-19 infections count in Tokyo, Japan, from January 16, 2020, to September 30, 2022.

Figure 1 illustrates the daily COVID-19 infection count in Tokyo during our sampling period, marked by seven distinct transmission waves. Furthermore, the spread of COVID-19 demonstrates non-linearity due to external factors, such as the implementation of public health and social measures recommended by the World Health Organization to curb the disease’s proliferation. Table 1 records seven waves of preventative measures issued by the Japanese government during our analyzed period. Evidently, these emergency measures effectively suppressed the transmission of COVID-19, as indicated by the successive decline of all waves during this period.

**Table 1:** The identified periods with significant fluctuation.

Wave	Start time	End time	Main issues	Government measures
1st	Jan 2020	Jun 2020	The rapid increase in positive cases and close contact cases with unknown infection routes	Held the Tokyo Metropolitan Crisis Management Council, citizens reduce going out, close facilities and restaurants, and shorten operating hours
2nd	Jul 2020	Oct 2020	The number of cases in restaurants and other entertainment venues in the “nightlife area” of the city center has surged	Request for reduce businesses hours, shorten the operating hours of liquor restaurants and karaoke venues
3rd	Oct 2020	Feb 2021	Increased risk of severe illness in the older adults and increased percentage of infections at year-end family gatherings	Declaration of a state of emergency by the government, residents who do not need to leave their homes and restaurants to shorten business hours
4th	Mar 2021	Jun 2021	An increase in the number of young people infected with the more infectious Alpha variant of the virus	Called for businesses to close temporarily or reduce operating hours, restaurants where residents are not required to leave their homes and provide alcoholic beverages are closed
5th	Jul 2021	Oct 2021	More middle-aged and young people are infected with Delta variant strains that can cause severe illness	Vaccination rate (ages 12 and over, 1st shot: 47.36%, 2nd shot: 36.66%), reduce 50% of employees going out and entering the office by 70%
6th	Jan 2022	May 2022	Increased infection with Omicron variant and severe illness in children and older adults households	Called for businesses to reduce operating hours, the number of table mates in the restaurant is limited to 4 and the stay time is limited to 2 h or less
7th	Jun 2022	Oct 2022	Omicron BA. 5 variant is more infectious	Vaccination rate (1st shot: 87.8%, 2nd shot: 87.3%, 3rd shot: 8.1%), coexistence policy with viruses, no new restrictions imposed on activities

Specifically, these seven waves of preventative measures included:

The first wave (January 2020–June 2020) saw a sudden spike in positive cases and close contact cases with unknown transmission routes. Urgent governmental measures included held the Tokyo Metropolitan Crisis Management Council, a temporary suspension of all schools, curtailment of unnecessary and non-emergency outings for urban residents, enterprise facility closures and shortened restaurant operating hours (5:00 am–8:00 pm), and the cancelation of events. These measures reduced interpersonal contact by 80% and significantly controlled the outbreak.

The second wave (July 2020–October 2020) recorded a case surge primarily linked to restaurants and entertainment venues in the city center’s “nightlife areas,” leading to a rise in infections among young people. In response, the government mandated request for reduce businesses hours, shortened operating hours for restaurants and karaoke venues that serve alcoholic beverages (5 am–10 pm), reducing nightlife activity and thus new cases without a state of emergency declaration.

The third wave (October 2020–February 2021) noted a substantial increase in infections compared to the first two waves. Unlike the second wave, infections at home were more prevalent. Consequently, the government declared a second state of emergency, necessitating restaurants and similar venues to reduce their operating hours (11 am–7 pm for alcohol service), limit unnecessary outings, curtail activities, and expedite vaccination efforts for the older adults and general public.

The fourth wave (March 2021–June 2021) was largely attributed to a more infectious Alpha variant, predominantly affecting younger generations. The government initiated a “stay-at-home week,” restricting inter-county travel for non-essential purposes, enforcing operational cessation or shortened operating hours for large facilities, mandating spectator-less activities, and limiting restaurant hours (5 am–8 pm).

The fifth wave (July 2021–October 2021), fueled by the highly virulent Delta variant, showed a rapid progression with a notable increase in new positive cases, particularly among those aged 40–60. A state of emergency was declared, implementing measures such as limiting non-essential travel, reducing footfall in crowded areas by 50%, ceasing operations for restaurants serving alcoholic beverages, shortening operating hours for other restaurants (5 am–8 pm), encouraging a 70% reduction in office workers via WFH, and promote the 2nd shot vaccination process of COVID-19.

The sixth wave (January 2022–May 2022), driven by the most infectious COVID-19 strain (Omicron variant), resulted in a surge in new cases, notably among children and the older adults. In response, the government issued a quasi-emergency state, called for businesses to reduce operating hours, limiting restaurant patrons to four per table (eight after April 25th), and restricting dining time to 2 h (non-certified restaurants were only allowed to serve drinks before 9 pm).

The seventh wave (June 2022–October 2022), characterized by a more infectious BA.5 strain replacing the Omicron variant BA.2, registered a surge in new positive cases. Rather than enforcing restrictive measures, in addition to promoting the process of receiving the 3rd shot vaccine of COVID-19, the government decided to enhance infection prevention and strike a balance with socio-economic activities, advocating for coexistence with COVID-19.

## Results

### New components construction based on adaptive Fourier decomposition results

**Figure 2:**
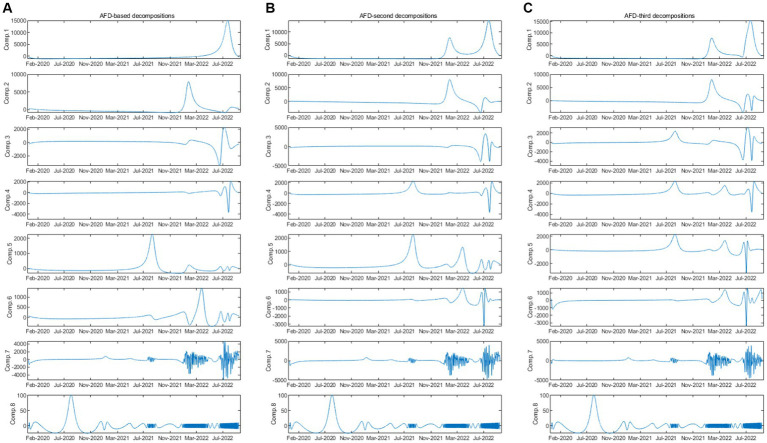
Different kinds of construction of the adaptive Fourier decomposition.

This section discusses the change process of the daily COVID-19 infection count under epidemic prevention policies in Tokyo by obtaining components at different time-frequency scales through adaptive Fourier decomposition (see Figure 2). The first to eighth vertical lines of a in the figure represent the frequency components from the lowest to the highest, reflecting changes in the number of infections at different scales, that is, changes from long-term to short-term. Similarly, b and c are in the vertical line also uses the (6) like a. The horizontal lines a to c in Figure 2 are the construction methods from the first to the third order when *q* = 1, 2 and 3 in (7), which reflect the changes in daily COVID-19 infection counts under the epidemic prevention policy under different AFD construction stages.

**Figure 3:**
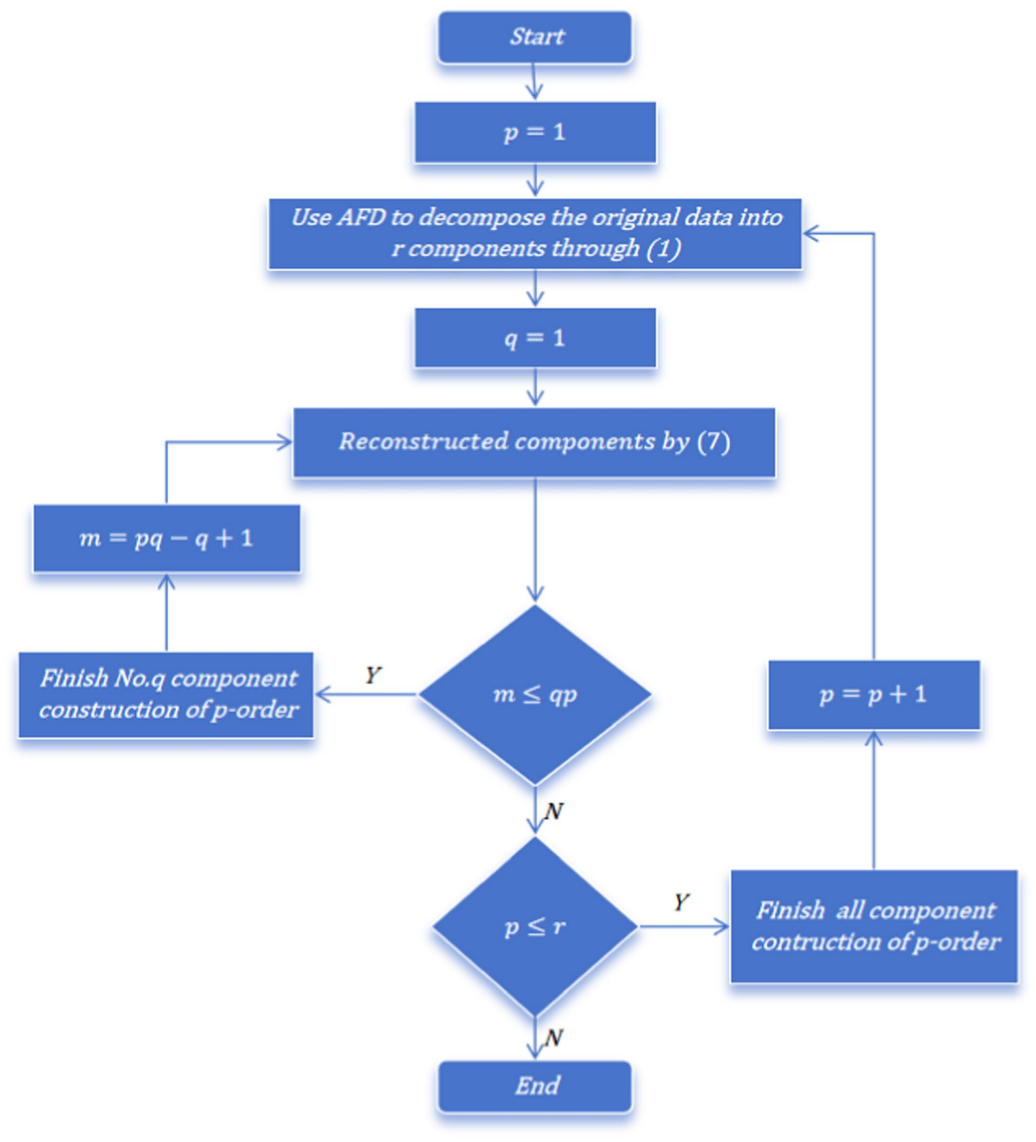
Construct new components flow chart of AFD decomposition.

Specifically, as shown in Figure 3, in the first-order decomposition, the original data is decomposed into 8 original components by AFD, which are then constructed into 8 new components based on the scenario where *q* = 1 in formula (7). As depicted in column a of Figure 2, Comp1 represents the component where *p* = 1 and *q* = 1, Comp2 represents the component where *p* = 2 and *q* = 1, and so on until Comp8 represents the component where *p* = 8 and *q* = 1. When these 8 new constructed components are superimposed using formula (8), the original signal is obtained.

In the second-order decomposition, the original data is first decomposed into 16 original components by AFD and then constructed into 8 new components according to *q* = 2 in formula (7). As shown in column b of Figure 2, Comp1 represents the component where *p* = 1 and *q* = 2, Comp2 represents the component where *p* = 2 and *q* = 2, and so on until Comp8 represents the component where *p* = 8 and *q* = 2. Similarly, when these 8 new constructed components are superimposed using formula (8), the original signal can be obtained.

For the third-order decomposition, the original data is decomposed into 24 original components by AFD. The 24 original components is then constructed by the scenario where *q* = 3 in formula (7), yielding 8 new constructed components. As illustrated in column c of Figure 2, Comp1 represents the component where *p* = 1 and *q* = 3, Comp2 represents the component where *p* = 2 and *q* = 3, and so forth until Comp8 represents the component where *p* = 8 and *q* = 3. Once again, when these 8 new constructed components are superimposed using formula (8), the original signal is recovered.

### The components derived using AFD

As shown in Figure 2 (longitudinal), by comparing the first component with the original number of daily infections (Figure 1), it can be found that the first component generally presents a trend of the overall number of infections during the sample period, indicating that the Tokyo government has held the Tokyo Metropolitan Crisis Management Council, declared a state of emergency and measures to reduce human contact are effective control of the epidemic. From the second to the seventh component, the Delta strain will appear in Tokyo from mid-2021 and the new positive cases will rise sharply, corresponding to the impact of the fifth to seventh waves (Table 1), and the impact generally reflects in the relatively medium and long-term number of infections. It can be understood that measures such as reducing the number of employees entering the office, shortening restaurant business hours, and promote the vaccination process of COVID-19 can effectively control human contact.

In contrast, the results of the eighth component show sharp fluctuations since 2020. These include spikes in the first to fourth waves, and the shocks of the fifth to seventh waves are absorbed at a relatively high frequency, that is in the medium and short term. It shows that early measures of held the Tokyo Metropolitan Crisis Management Council and its policies of declared a state of emergency and request for reduce businesses hours that let the COVID-19 has been very effectively controlled. For the eighth component with the highest frequency, severe fluctuations can be observed from the fifth wave to the seventh wave, reflecting the short-term local fluctuations during the shock. The reason why the spread of the COVID-19 has been slowed down is that control measures have been taken, such as promoting people of different ages to be vaccinated the 2nd shot and 3rd shot of COVID-19.

### The new construction components base on AFD

As shown in Figure 2 (horizontal), by comparing the first-order, second-order and third-order new construction components result (Figures 2A–C). It can be observed that in the first component, the second-order and third-order AFD new construction components shows a better overall trend in the number of infected individuals compared to the first-order new construction components, and can initially reflect the daily number of infections under the entire epidemic prevention measures. From the second to seventh components, the third-order new construction components results better reflect the data situation of the daily COVID-19 infection count under epidemic prevention policies in the mid-term. Further discuss the correlation between the new construction components result and the daily COVID-19 infection count under epidemic prevention policies, as well as the relationship between different new construction components result.

As shown in Table 2, we conducted Pearson correlation analysis on the decomposition results and analyzed the correlation between the data results within a confidence interval of *p* < 0.05. The coefficients of the first component are much higher than those of the other components, and the coefficients of each component from low frequency to high frequency also decrease, which is basically consistent among the components of the new construction components result of each order, These correlations reflect the relationship between the information contained in the component and the daily COVID-19 infection count. The higher the correlation between a specific component and the original number of infections, to a certain extent, it means that the component can better explain the fluctuations in the daily COVID-19 infection count. According to statistics, except for the eighth component, the other components are positively correlated with the daily COVID-19 infection count under epidemic prevention policies at a significance level of 1%.

**Table 2:** The correlations between components of distinct new construction components and original infections.

	Correlations between components and original infections			Correlations between components of distinct methods		
	AFD-based	AFD-second	AFD-third	AFD-based&AFD-second	AFD-based&AFD-third	AFD-second&AFD-third
Comp. 1	$0.840^{***}$	$0.938^{***}$	$0.955^{***}$	$0.895^{***}$	$0.879^{***}$	$0.982^{***}$
	(0.000)	(0.000)	(0.000)	(0.000)	(0.000)	(0.000)
Comp. 2	$0.418^{***}$	$0.455^{***}$	$0.472^{***}$	$0.918^{***}$	$0.885^{***}$	$0.964^{***}$
	(0.000)	(0.000)	(0.000)	(0.000)	(0.000)	(0.000)
Comp. 3	$0.180^{***}$	$0.222^{***}$	$0.250^{***}$	$0.818^{***}$	$0.721^{***}$	$0.880^{***}$
	(0.000)	(0.000)	(0.000)	(0.000)	(0.000)	(0.000)
Comp. 4	$0.126^{***}$	$0.173^{***}$	$0.190^{***}$	$0.729^{***}$	$0.666^{***}$	$0.913^{***}$
	(0.000)	(0.000)	(0.000)	(0.000)	(0.000)	(0.000)
Comp. 5	$0.118^{***}$	$0.141^{***}$	$0.160^{***}$	$0.837^{***}$	$0.740^{***}$	$0.884^{***}$
	(0.000)	(0.000)	(0.000)	(0.000)	(0.000)	(0.000)
Comp. 6	$0.077^{***}$	$0.108^{***}$	$0.130^{***}$	$0.718^{***}$	$0.596^{***}$	$0.830^{***}$
	(0.000)	(0.000)	(0.000)	(0.000)	(0.000)	(0.000)
Comp. 7	$0.222^{***}$	$0.209^{***}$	$0.196^{***}$	$0.942^{***}$	$0.880^{***}$	$0.938^{***}$
	(0.000)	(0.000)	(0.000)	(0.000)	(0.000)	(0.000)
Comp. 8	0.006	0.006	0.006	$1.000^{***}$	$1.000^{***}$	$1.000^{***}$
	(0.417)	(0.417)	(0.417)	(0.000)	(0.000)	(0.000)

^***^p < 0.01, ^**^p < 0.05, ^*^p < 0.1.

The correlation coefficients are all estimated by Pearson’s correlation method.

The results show that each order is significantly correlated at 1%, which means that the AFD decomposition and new construction components result are strongly correlated, and the coefficient of the first component of the third-order is 0.955, which is much larger than the other seven components. The coefficient generally decreases with the increase of frequency. These results highlight the dominance of the lowest frequency component that reflects the trend, among which the low frequencies in the third-order new construction components result better reflect the fluctuations of the original data.

### Comparison with other time-frequency methods

**Figure 4:**
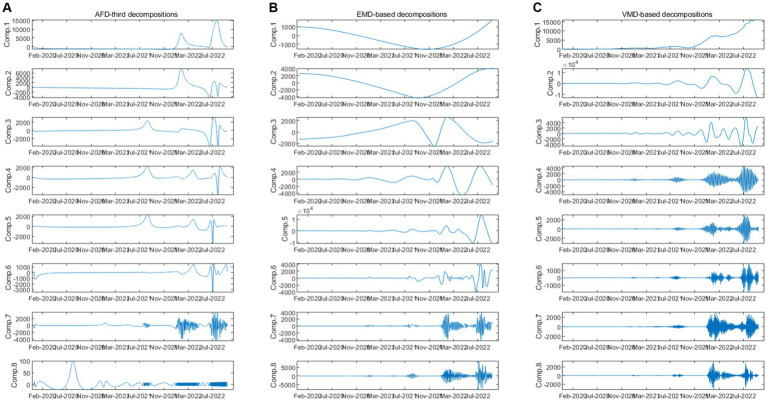
Decompositions based on AFD-third, EMD, and VMD.

We then compare the results obtained from AFD with two commonly used time-frequency methods, including EMD and VMD. The decomposition results of the two methods are shown in Figures 4B,C, and the correlation between the components and the daily COVID-19 infection count under epidemic prevention policies is shown in Table 3. It can also be seen from Figure 4 that the first component obtained from VMD is similar to that obtained from AFD, and the coefficients are all much higher than the other components. Meanwhile, the fourth to sixth components obtained from EMD have similar peaks to those obtained from AFD. In addition, as the frequency increases, the coefficients of each component of VMD also decrease, which is consistent with the results obtained by AFD decompositions.

**Table 3:** The correlations between components of different method and original infections.

	Correlations between components and original infections			Correlations between components of distinct methods		
	AFD-third	EMD-based	VMD-based	AFD-third&EMD-based	AFD-third&VMD-based	EMD-based&VMD-based
Comp. 1	$0.955^{***}$	$0.124^{***}$	$0.752^{***}$	$0.177^{***}$	$0.727^{***}$	$0.179^{***}$
	(0.000)	(0.000)	(0.000)	(0.000)	(0.000)	(0.000)
Comp. 2	$0.472^{***}$	$0.282^{***}$	$0.650^{***}$	$-0.014^{*}$	$0.422^{***}$	$0.014^{**}$
	(0.000)	(0.000)	(0.000)	(0.054)	(0.000)	(0.046)
Comp. 3	$0.250^{***}$	−0.023	$0.301^{***}$	$0.317^{***}$	$0.369^{***}$	$0.029^{***}$
	(0.000)	(0.465)	(0.000)	(0.000)	(0.000)	(0.000)
Comp. 4	$0.190^{***}$	$0.513^{***}$	$0.160^{***}$	$-0.028^{***}$	0.010	0.001
	(0.000)	(0.000)	(0.000)	(0.000)	(0.160)	(0.901)
Comp. 5	$0.160^{***}$	$0.562^{***}$	$0.110^{***}$	$0.163^{***}$	$0.039^{***}$	−0.001
	(0.000)	(0.000)	(0.001)	(0.000)	(0.000)	(0.897)
Comp. 6	$0.130^{***}$	$0.236^{***}$	$0.069^{**}$	$0.070^{***}$	$0.017^{**}$	$0.016^{**}$
	(0.000)	(0.000)	(0.03)	(0.000)	(0.013)	(0.020)
Comp. 7	$0.196^{***}$	$0.150^{***}$	$0.103^{***}$	$0.706^{***}$	$0.364^{***}$	$0.075^{***}$
	(0.000)	(0.000)	(0.001)	(0.000)	(0.000)	(0.000)
Comp. 8	0.006	$0.334^{***}$	$0.083^{***}$	0.004	0.005	$0.353^{***}$
	(0.417)	(0.000)	(0.009)	(0.588)	(0.442)	(0.000)

^***^p < 0.01, ^**^p < 0.05, ^*^p < 0.1.

The correlation coefficients are all estimated by Pearson’s correlation method.

To further discuss the correlation between the decomposition results of AFD, EMD and VMD, we also estimated the Pearson correlation between the components obtained by these models. Interestingly, each component of AFD was positively correlated with EMD and VMD at the 5% significance level for 75% of components, while the correlation coefficients among the remaining components were not significant. This indicates that AFD is highly correlated with most components of the other two methods. Most of the coefficients of the EMD and VMD components are insignificant, it means that the decompositions between EMD and VMD are weakly correlated. Therefore, AFD performed best in absorbing useful information about the effectiveness of epidemic prevention measures obtained from these methods.

### Connection between AFD decompositions and government measures

**Figure 5:**
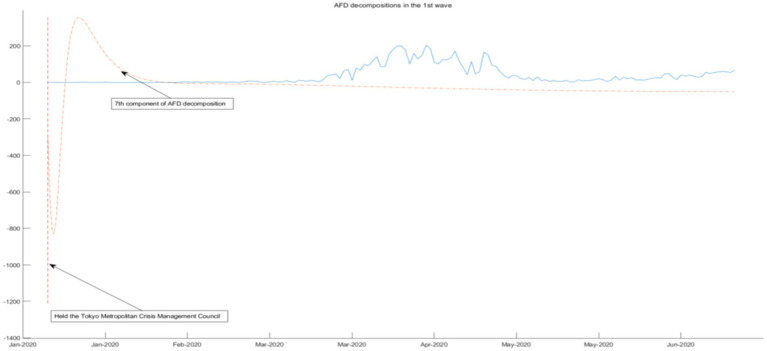
AFD decomposition components in the 1st wave.

**Figure 6:**
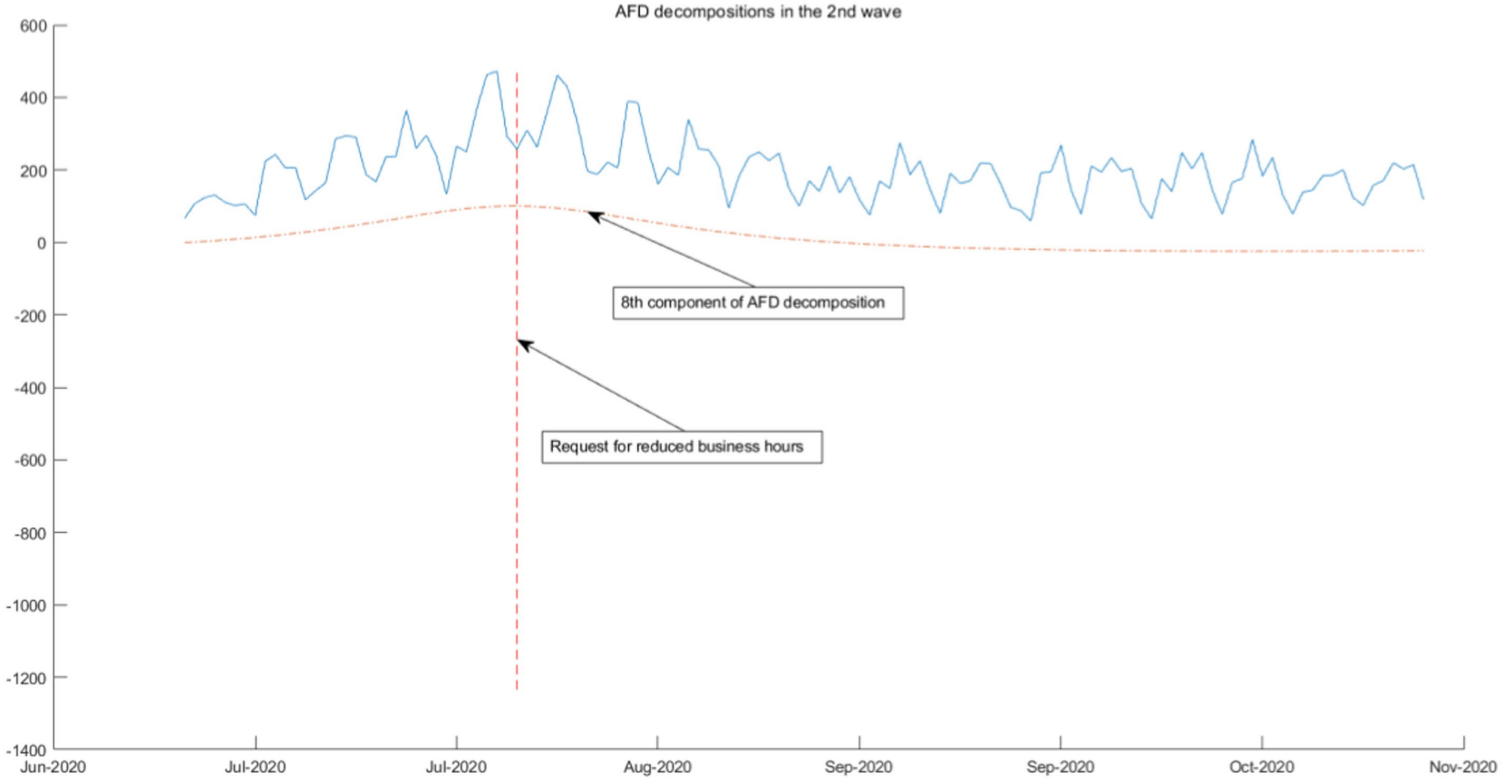
AFD decomposition components in the 2nd wave.

**Figure 7:**
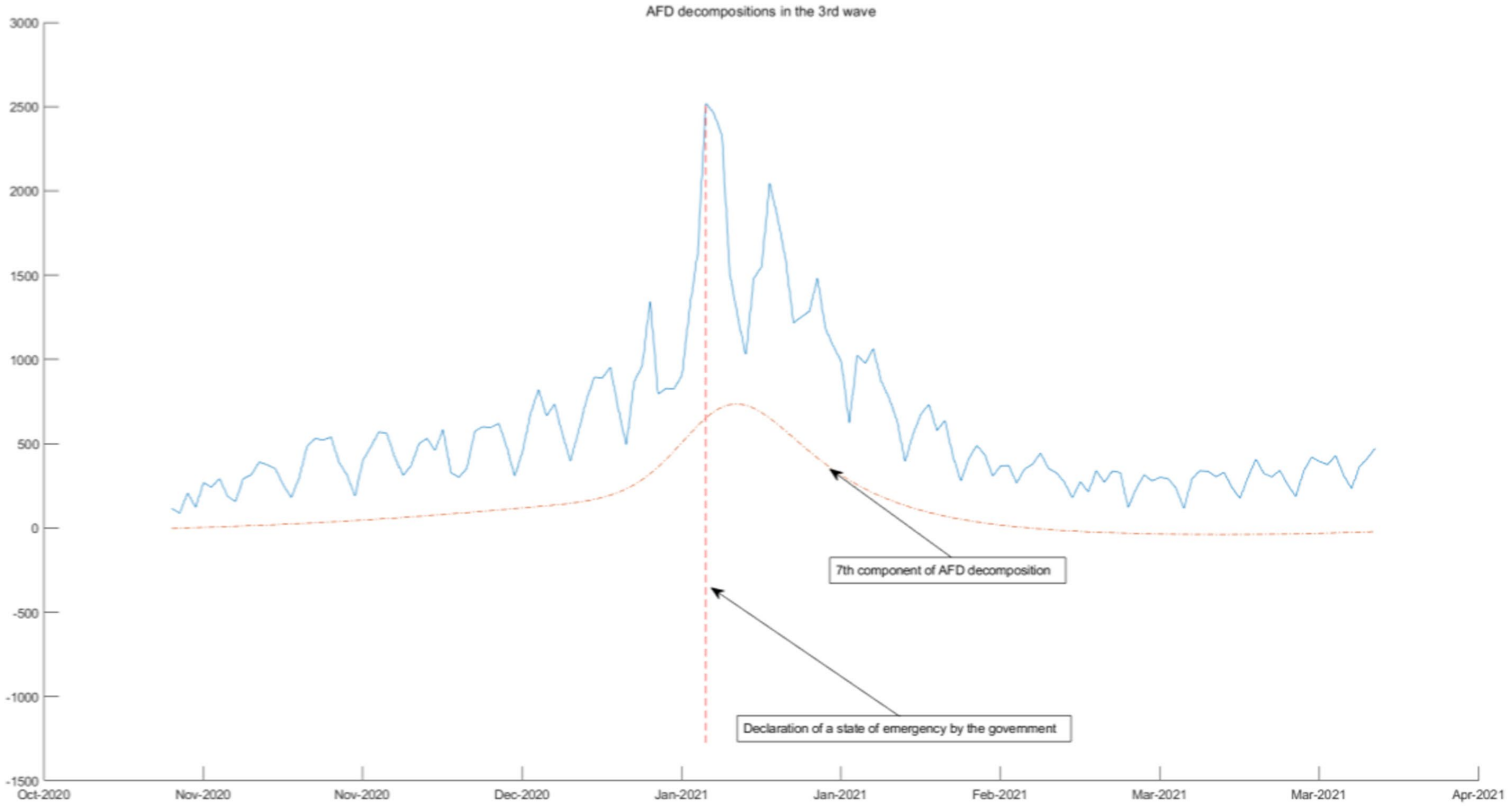
AFD decomposition components in the 3rd wave.

**Figure 8:**
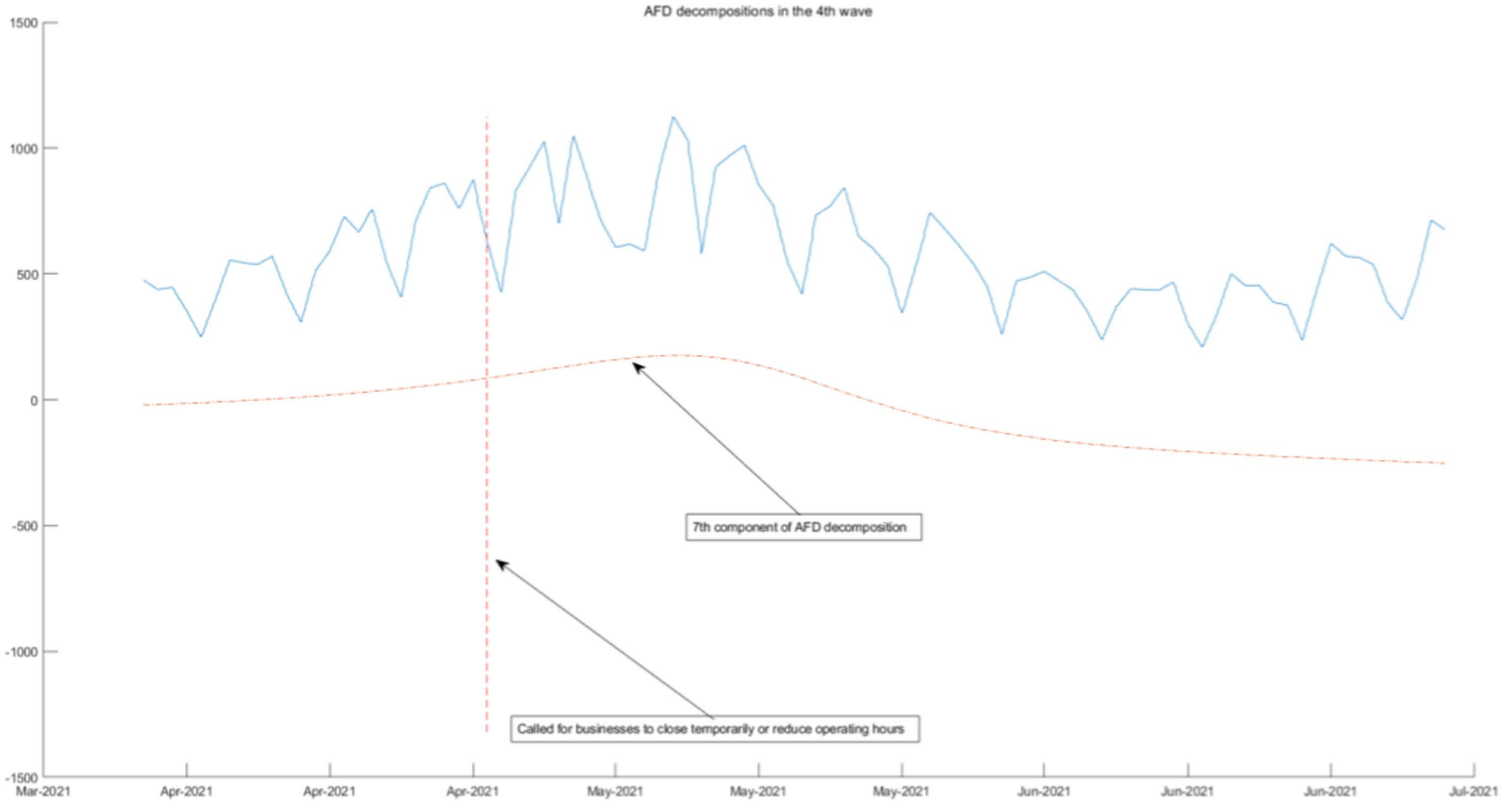
AFD decomposition components in the 4th wave.

**Figure 9:**
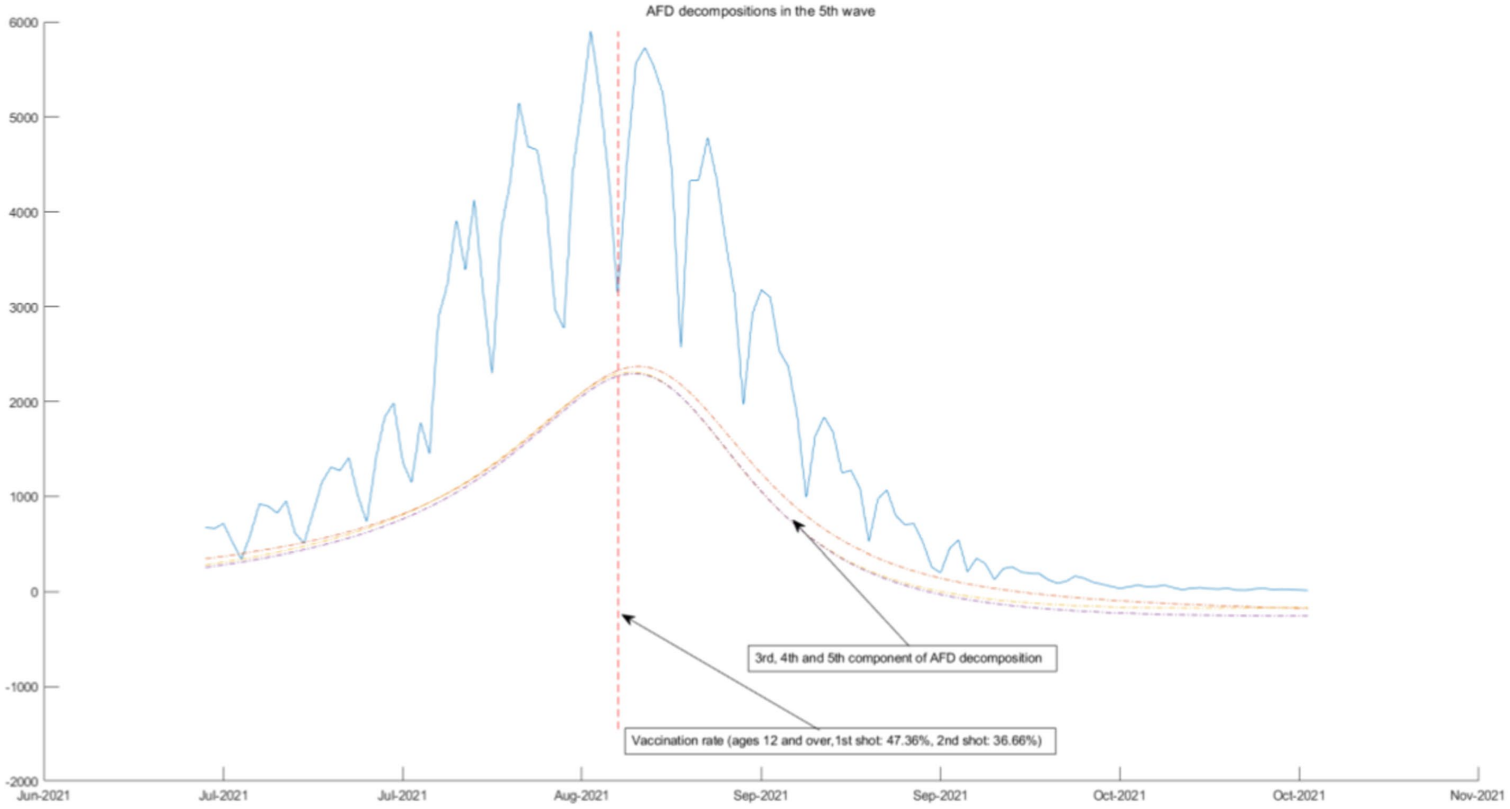
AFD decomposition components in the 5th wave.

**Figure 10:**
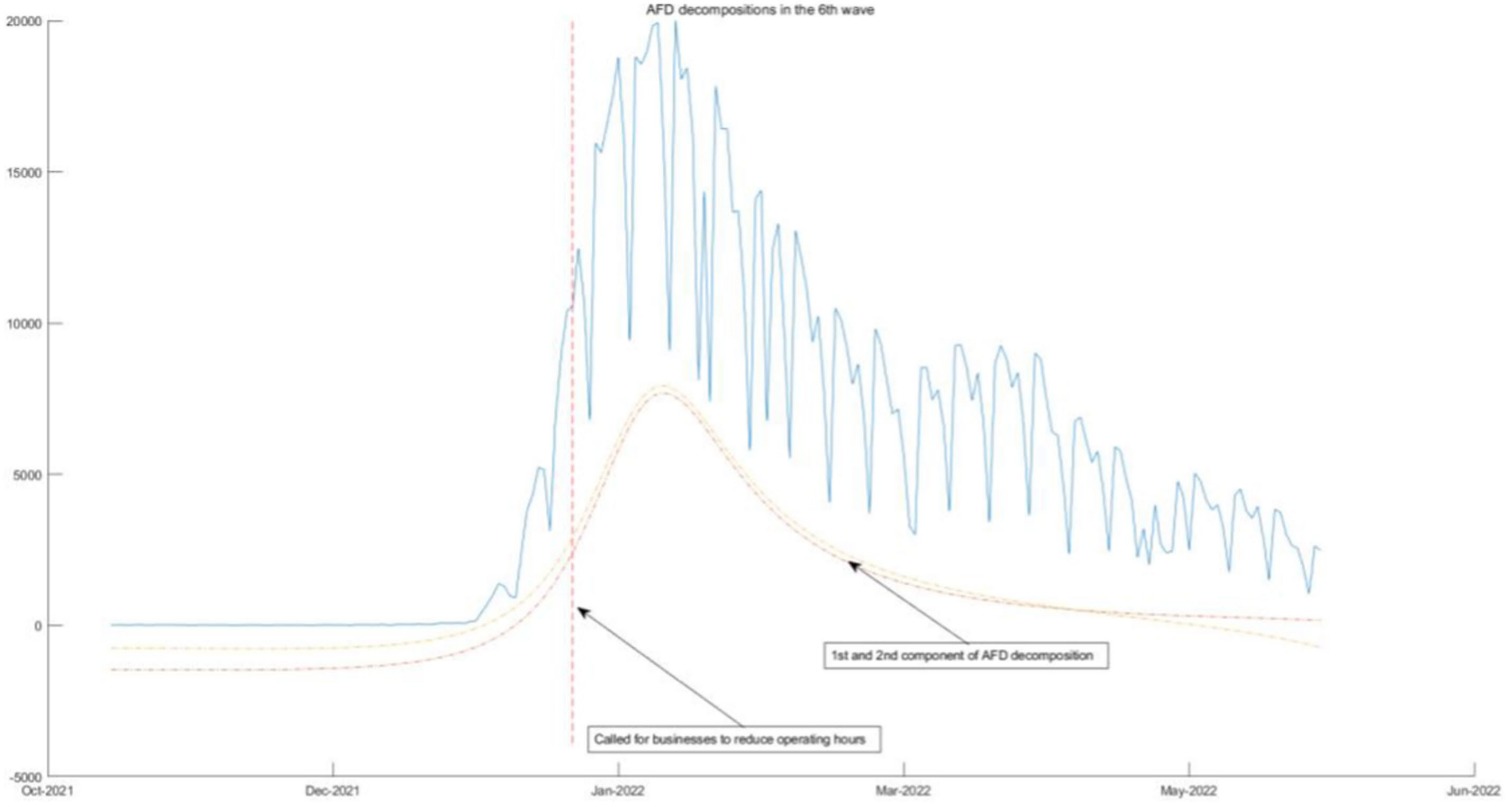
AFD decomposition components in the 6th wave.

**Figure 11:**
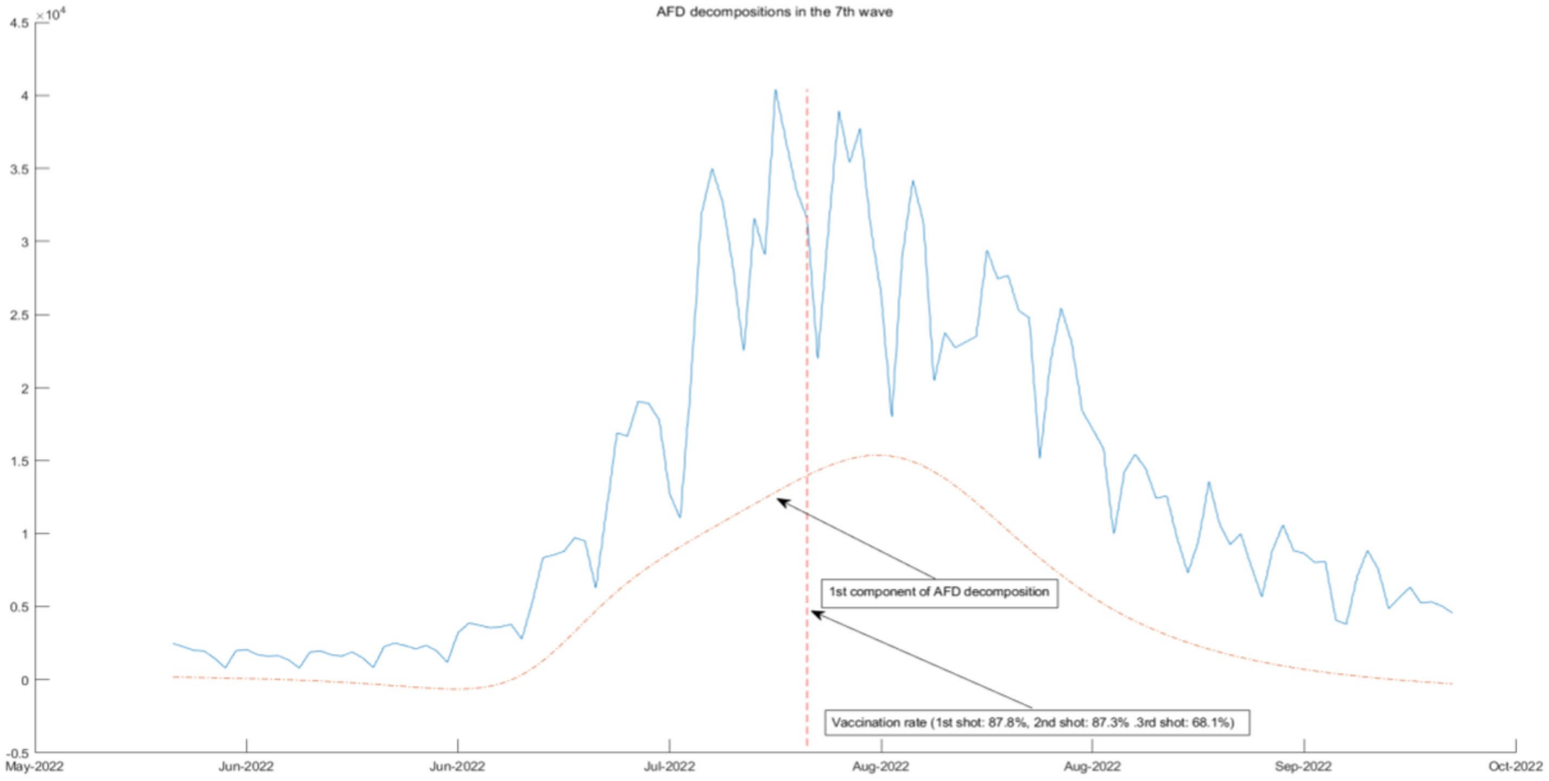
AFD decomposition components in the 7th wave.

We use the 3rd-order AFD new construction components result and the daily COVID-19 infection count under epidemic prevention policies to segmentation and combination. According to the time interval of the seven-wave epidemic in Tokyo, it can be divided into seven segmented graphs from Figures 5–11, which annotate the components that can be used to explain the prevention policies and new construction components associated with the daily COVID-19 infection count in the segmented graphs. The components shown in the figure have correlation coefficients greater than 0.7 at a 5% confidence interval.

As shown in Figure 5, in the face of unprecedented crises and challenges under the epidemic and widespread attention to unknown viruses, the Tokyo Metropolitan Crisis Response Conference was held, and a formation and helpline policy was prepared at the City Hall. From the aspects of epidemic situation, epidemic spread, and epidemic prevention, it can be seen from the seventh component that this policy state has released the energy of this wave of epidemic in advance, effectively suppressing the spread of COVID-19, and thus reducing all waves during the first wave of the epidemic. Other policies, including partial cancelation and measures to prevent the spread of the disease, have also been used to gradually control the spread of COVID-19.

As the epidemic was brought under control, the first wave of the epidemic came to an end, policies were gradually relaxed, and people returned to normal lives. As shown in Figures 6–8, from June 2020, Tokyo experienced the second, third, and fourth waves of the epidemic, and policies calling for businesses to shorten their business hours were also implemented. From the seventh and eighth components of the AFD decomposition results during this period, this policy effectively releases the energy of the epidemic, and the daily COVID-19 infection count individuals quickly decreases. As shown in the figure, the policy is very effective, indicating that reducing people’s time spent in the same space is an effective measure. This is because the seventh and eighth components are high-frequency components, that means that the energy changes very quickly, so it can be inferred that the policies calling for businesses to shorten their business hours were effectiveness is high.

The above four waves of COVID-19 are all related to one component of AFD. In Figures 9, 10, we will see that the COVID-19 policy is related to multiple components of AFD. This indicates that the energy of the fifth and sixth waves of COVID-19 is greater than the first three waves, and the daily peak number of infected individuals will be higher. As shown in Figure 9, the fifth wave of epidemic policies is related to the frequency component of AFD decomposition results, which means that the effect of the two-dose vaccination policy on the epidemic is moderate and needs to be sustained for a long time. The sixth wave of the epidemic in Figure 10 once again calls for enterprises to reduce their business hours, which is related to the first and second components of the AFD decomposition results. This is because the long-term implementation of this policy has affected the normal operation of enterprises, so the policy effect is not as good as before. Therefore, the sixth wave is no longer related to the seventh and eighth components of the high frequency, but instead to the first and second components of the low frequency, indicating that the effects of the same policy are different in different periods.

In Figure 11, the seventh wave of epidemic policies is associated with the first component of AFD decomposition results, which is the lowest frequency component. The three-shot vaccination policy is effective in this epidemic, but its effectiveness is very limited and cannot be seen in a short period of time. Therefore, the three-shot vaccination policy should be persisted in the long term.

Based on the above, among the various policies implemented by the government during several waves of epidemics, some policies are related to the high-frequency decomposition results of AFD, some are related to the low-frequency decomposition results, and some are not related, which is related to the effectiveness of specific policies. The policy of calling on enterprises to reduce business hours is a very effective policy that can quickly control the epidemic in a short period of time, as it is related to the seventh and eighth components of the high-frequency decomposition results of AFD in the first four waves of the epidemic; However, due to other factors in reality, the policy of the sixth and seventh wave of COVID-19 is related to the first and second components of the low-frequency decomposition results of AFD, and even the policy of the seventh wave of COVID-19 is no longer related to the decomposition results of AFD, indicating that the policy is gradually losing its effectiveness. The fifth wave of the epidemic is that the vaccination policy has played a good role, with two doses of vaccine reaching the third, fourth, and fifth components of the AFD decomposition results; By the seventh wave of the epidemic, the policy of receiving three doses of vaccine has also been reduced to be related to the low-frequency first component of AFD decomposition results. This indicates that the vaccination policy can still effectively reduce the daily COVID-19 infection count caused by the epidemic, but the effectiveness of implementation will gradually weaken.

## Discussion

In this article, we carefully studied the fluctuation of the daily COVID-19 infection counts in Tokyo and the various epidemic prevention measures implemented by the government, in order to find out which policies are most effective in influencing the epidemic. Given the representativeness of Tokyo’s epidemic prevention measures, our empirical analysis mainly focuses on this city. We not only utilized AFD to decompose the daily COVID-19 infection counts under epidemic prevention policies, but also constructed the multi-level composite components composed of the decomposition results, thus extracting elements of different combination scales. Our analysis of the daily number of infections showed that there were seven periods of significant fluctuations throughout the entire study period, mainly due to request for reduce businesses hours and implement vaccination policies. In addition, from the new construction components at different combination scales, we found that the higher the frequency of AFD component and the daily COVID-19 infection counts under policies, the greater the Pearson coefficient and stronger the correlation. With the continuous implementation of policies, the correlation between policies and high-frequency components of AFD decomposition results has gradually shifted to low-frequency components, indicating that the effectiveness of policies is decreasing. Comparing the decomposition results with the results of EMD and VMD shows that AFD is the most effective in absorbing valuable information on time-domain and frequency-domain epidemic prevention measures obtained by the three methods.

In summary, using AFD method to analyze epidemic data may provide a meaningful reference point for future public health policy and social measure formulation.

## Data availability statement

Publicly available datasets were analyzed in this study. This data can be found here: https://covid19.mhlw.go.jp/public/opendata/newly_confirmed_cases_daily.csv.

## Author contributions

CH designed the study. GL and ZiL collected and managed the data. GL, CH, and WH reviewed the literature. TQ, CH, and WQ developed the methods. GL analyzed the data. GL and ZhL drafted the manuscript. All authors discussed the results and contributed to the final manuscript, read, and agreed to the published version of the manuscript.
